# Over 25 Years of Hope: Development of Lymphatic Filariasis Patient Support Groups in Haiti

**DOI:** 10.4269/ajtmh.23-0607

**Published:** 2024-04-23

**Authors:** Valery Madsen Beau De Rochars, Jeannine Coreil, Martha Désir, Gladys Mayard, Marie Carmel Michel, Marie Denise Milord, Rand Carpenter, Thomas G. Streit, Luccène Désir, Gregory S. Noland, David G. Addiss

**Affiliations:** ^1^ Department of Health Services Research, Management and Policy, College of Public Health and Health Professions, University of Florida, Gainesville, Florida;; ^2^ Emerging Pathogens Institute, University of Florida, Gainesville, Florida;; ^3^ The Carter Center, Atlanta, Georgia;; ^4^ Hôpital Sainte Croix, Leogane, Haiti;; ^5^ Department of Community and Family Health, College of Public Health, University of South Florida, Tampa, Florida;; ^6^ Centre de Recherche et Service Social Humanitaire, Port-au-Prince, Haiti;; ^7^ Mennonite Central Committee, Akron, Pennsylvania;; ^8^ IMA World Health, Washington, District of Columbia;; ^9^ University of Notre Dame, South Bend, Indiana;; ^10^ Focus Area for Compassion and Ethics, The Task Force for Global Health, Decatur, Georgia

## Abstract

Support groups can create environments that are conducive to healing and well-being, particularly for persons with stigmatizing chronic diseases. In 1998, the support group concept was adapted in Haiti for persons with disabling lymphedema caused by lymphatic filariasis (LF). The project was developed with the expectation that the support group model conceived in the developed world be interpreted and modified by persons affected with lymphedema in the Haitian setting. Initiated with modest financial support within a research initiative to eliminate LF, a total of 50 “Hope Clubs” were formed from 1998 to 2023 across seven communes (districts) located in 3 of Haiti’s 10 regional Departments. Documented benefits of the support groups included improved limb self-care, decreased incidence of inflammatory episodes (adenolymphangitis), enhanced self-efficacy, economic benefit through microenterprise, and improved quality of life. Despite challenges of funding shortfalls, natural disasters, and political insecurity, persistence of LF support groups in Haiti highlights the crucial role of group ownership by affected persons and the freedom to reinvent the support group concept in light of local social, cultural, and economic conditions.

## INTRODUCTION

Lymphatic filariasis (LF) is a mosquito-transmitted neglected tropical disease (NTD) in low-income tropical and subtropical environments. The World Health Organization (WHO) aims to eliminate LF as a public health problem globally. The strategy includes interruption of transmission through mass drug administration (MDA) and care for those with LF through morbidity management and disability prevention (MMDP).

Although the majority of infected persons do not manifest overt disease, those who do can find themselves shackled to the bottom of the economic ladder, deprived of their potential, without medical care, and bereft of community support or concern. An estimated 40 million persons are disfigured by LF globally. Lymphedema after infection with *Wuchereria bancrofti,* the most common LF parasite, is more prevalent in women than in men,^1^^–^^3^ with a ratio in Haiti of approximately 7 to 1.^1^

Repeated bacterial skin infections exacerbate lymphatic dysfunction associated with LF, resulting in painful episodes of adenolymphangitis (ADLA), progression of lymphedema, and subsequent disability. With progression of lymphedema, bacterial and fungal overgrowth of the skin results in a foul odor. Affected people become outcasts, isolated from their communities and sometimes even from family. Indeed, the term “elephantiasis,” previously used in common parlance and professional publications to describe advanced lymphedema, conveys stigma. Consequently, the use of this term is now discouraged. Lymphatic filariasis is at once a consequence and a cause of poverty.^4^

In Western societies, peer support groups emerged from the self-help movement of the 20th century, with direct links to groups like Alcoholics Anonymous and WeightWatchers.^5^ Support groups also have been used to address the needs of people suffering from chronic illnesses such as AIDS, cancer, Alzheimer’s disease, and tuberculosis.^6^^–^^8^ Since the early 1980s, support groups have formed on the internet.

Remarkable progress has been made in interrupting LF transmission.^9^ But can resource-poor countries where filariasis is endemic, with inadequate health systems and shortages of health professionals, meet the needs of people who suffer from chronic filarial lymphedema? Our experience in Haiti suggests that this is indeed possible, with the involvement of affected persons and reliance on self-care, peer support networks, and patient education. We summarize our experience herein, much of it based at Hôpital Ste. Croix in Leogane, Haiti, which, for four decades, has served as a focus for research on *W. bancrofti* transmission control and MMDP programming.

In the 1990s, Gerusa Dreyer, a Brazilian physician, developed a simple regimen of hygiene, skin care, and physical therapy that dramatically reduced secondary infections, ADLA, swelling, and discomfort associated with filarial lymphedema.^10^ At the time, this approach was revolutionary, as the progression of filarial lymphedema was previously considered inevitable and irreversible. Dr. Dreyer showed that the regimen (consisting of washing with plain soap and water, movement, and elevation of the affected limbs) can be implemented at home and used in settings with minimal infrastructure and resources.

After training by Dr. Dreyer during the 1990s, the Hôpital Ste. Croix team received funding from the WHO’s Special Program for Research and Training in Tropical Diseases (TDR) to assess the effectiveness of this regimen. Studies between 1995 and 1998 demonstrated that adherence to treatment resulted in fewer ADLA episodes as well as decreased progression and histological improvement of lymphedema.^11^^,^^12^ Research from 2000 to 2002 showed that providing lymphedema patients with soap was associated with significant decreases in ADLA frequency.^13^ Based on this evidence of the feasibility and effectiveness of basic lymphedema management, as well as evidence from India and Brazil,^14^^,^^15^ the WHO recommended simple measures of hygiene and skin care as standard treatment of filarial lymphedema.^16^ A national reference center for lymphedema care was established in Leogane, and in 1995, additional outreach and clinics were established at Hôpital Sacré Coeur in Milot (North Department), with support from IMA World Health, the Mennonite Central Committee, and the Bill & Melinda Gates Foundation.

### Formation of LF support groups in Haiti.

Support groups in Haiti have historical roots dating back to the 19th century, including cooperative labor associations and grassroots peasant societies engaged in self-help and civic projects.^17^ When the lymphedema clinic was established at Hôpital Ste. Croix, however, there was no indigenous model for peer support groups focused on disease management in Haiti. Hospital staff were aware of the pioneering work by Dr. Dreyer in Brazil, who had organized “Hope Clubs”—assemblies of affected people and their families who come together to promote community awareness and understanding of lymphedema, as well as a more positive image of those living with the condition.^18^

In 1998, Jeannine Coreil, a medical anthropologist with experience in Haiti, initiated the local adaptation (“indigenization”) of the Hope Club concept and studied its impact.^19^ Women with lymphedema expressed an overwhelming preference for support groups to enable them to engage with other affected persons in mutual support.^20^ The goals of the research, supported by TDR, were to assess the applicability of a chronic disease support group model in Haiti, to describe the process of indigenization of the model, and to measure the impact of the intervention on patient quality of life and adherence to treatment.

Over a 2-year period, five support groups of 15 to 20 women each were organized in Leogane.^21^ Coreil and colleagues documented high levels of participation and enthusiasm and showed that the support group model had become indigenized to the Haitian cultural context, as reflected in the emphasis on spiritual and expressive components and training in practical skills. Support group participation provided benefits for quality of life, including less time spent managing the disease, less interference with work and family life, better understanding of the disease, improved adherence to home care practices, and fewer lymphedema-related symptoms.^21^

In 2000, a Presbyterian minister, Ruth Boling, obtained a grant from the Birthday Offering Fund of the Presbyterian Women, Presbyterian Church, USA, to continue and expand the program (2001–2003). The goals of the project were to replicate the support group intervention throughout the Leogane Commune and expand the program to other areas of Haiti where LF was endemic. In Leogane, 20 support groups were organized in 16 areas in accordance with the original model, reaching more than 400 women. Eight additional groups were organized in the municipalities of Archaie and Cabaret in 2004, while 17 groups were formed in four communes of the North Department (Limonade, Quartier Morin, Milot, and Plaine du Nord) from 2000 to 2003. In 2005, the support group model expanded to other departments in Haiti, including Port-de-Paix in the Northwest Department, where it was integrated into the community health program. Five support groups were created there, with more than 100 participants.

In 2005, Gladys Mayard, a Haitian anthropologist, developed a guide and educational materials to train support group coordinators and facilitators in the new localities.^22^ Support groups met weekly, with an agenda adapted by the participants. Each support group was served by a part-time animator (typically one of the patients tasked with administrative and logistical duties). The animator was also responsible for visiting group members at home, assessing the quality of lymphedema self-management as well as quality of life, identifying unmet needs, and encouraging family members to get involved with their loved one’s care. The animator kept the clinic informed about patient status between clinic visits and provided basic supplies for hygiene. She also served an on-call function to assist members with questions or in the rare event of ADLA.

Although the character, interpersonal “chemistry,” and ebb and flow of each group were distinct, a general pattern for the meetings developed wherein prayer and singing were often followed by individual sharing and updates on health status, some role-playing, review of home-care treatment protocols or a particular aspect of LF, and perhaps a visit by an expert seamstress or craftsperson who could teach new skills or guide patients to new levels of artistry. Cultural discussions were popular with the groups, with refreshments and paid outside speakers invited when funds were available. A few groups with less-engaged members met less frequently, and interpersonal conflicts led to the temporary dissolution of two support groups.

The educational component of the support groups incorporated a set of 10 cassette tapes developed by Maude Heurtelou, a Haitian-American consultant, with separate funding from the Office of Women’s Health of the CDC. Each tape introduced an important topic related to LF transmission and management, using culturally appropriate story lines and characters and focusing on home care practices, destigmatization of the illness, and family and community support. An instructor’s manual and illustrated patient booklets accompanied the tapes. In addition, an original song was composed and recorded to accompany the tapes, with each lesson introducing new lyrics related to the educational content. The song became known as the “Filariasis Song” and was broadcast, along with the tapes and other educational messages, by the Hôpital Ste. Croix community radio station, garnering attention for the plight of LF-affected families and reinforcing the basic principles of lymphedema care. Subsequent years saw the expansion of the support group model, the continuity of group functioning during subsequent periods of resource scarcity, and the integration of support groups into local and national filariasis control programs.

**Figure 1.**
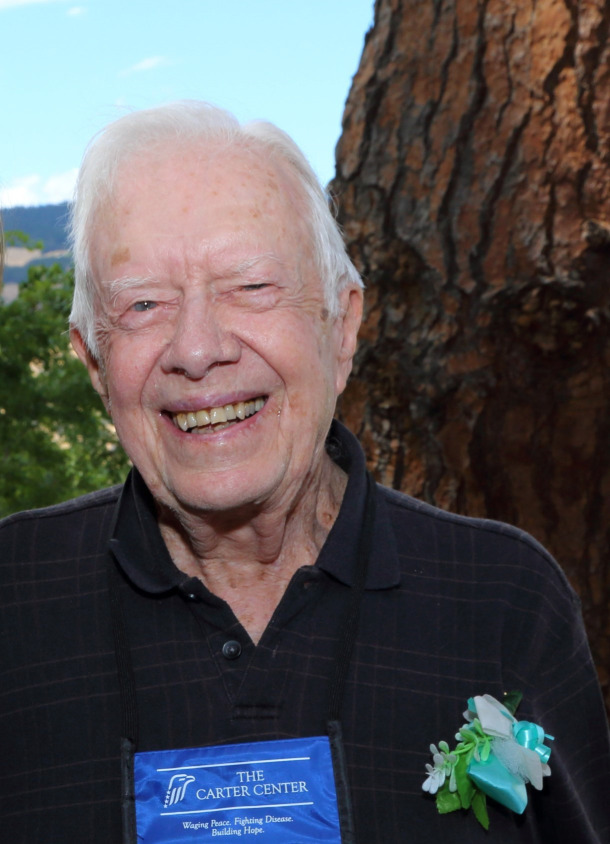
President Carter wears a boutonnière made by women from the Leogane, Haiti, Vocational Center for Patients with Filariasis.

One component of the support group program that grew in significance over time was instruction in making artificial flowers and other crafts, which participants could sell to supplement their income (Figure 1). Feedback from support group members underscored the importance to them of gaining such income-generating skills and the need for a venue to sell craft goods. Gladys Mayard and her team received a grant from Self-Development of People, a ministry of the Presbyterian Church, to establish a microenterprise project run by the patients. The project, funded from 2004 to 2006, included the renovation of a local house to serve as a community store where women sold flower arrangements and other crafts. The women also gained skills in business administration, budget planning, marketing, and accounting. These activities were extended in 2014 as a vocational center was established.

The NTD community has increasingly recognized the mental health burden of LF and other NTDs.^23^^,^^24^ Depression, often severe, is a common finding among persons with filarial lymphedema.^24^ In 2019, The Carter Center piloted a week-long Chronic Disease Self-Management program in Leogane to support the mental and emotional well-being of Hope Club members.^25^ The prevalence of self-reported depressive illness decreased from 50% at baseline to 28% at the end of the study, although results were not statistically significant because of the small sample size and loss to follow-up.^26^

The support group model pioneered in Haiti has spread to other countries and is now recommended by the WHO as an essential component of MMDP.^16^ In Nigeria, LF support groups initiated by The Carter Center continue to expand.^27^

### Challenges.

Clinical and social services for persons with filarial lymphedema have evolved over the past 25 years, beginning with research to confirm the effectiveness and feasibility of hygiene and skin care, expanding with formative research on support groups, and continuing with the establishment of clinics for lymphedema care and social support in the form of support groups and Hope Clubs. Funding for these activities has been inconsistent and often inadequate, even though the costs for clinics and support groups are relatively low. For example, the lymphedema clinic and the associated Hope Clubs in the North Department closed in 2007 because of funding shortages. Funding shortfalls resulted in temporary suspensions of the project in Leogane during 2006 and again from 2014 through 2015. Over time, pragmatic concerns and ever-present financial pressures on Haitian patients and their families led to adjustment of support group content, with a greater focus on microenterprise opportunities and less emphasis on processing feelings about the illness. In addition to disease-specific challenges, Hope Club members also suffer the effects of natural disasters (earthquakes and hurricanes) and crippling political instability and insecurity that confront the general Haitian population. Yet even with these serious setbacks, support groups have continued.

## CONCLUSION

Planners for health programs in resource-poor settings often limit their focus to institutional facilities, as these are the traditional locus of clinical and public health activities. Our experience with LF in Haiti underscores the value of community-based interventions, especially low-cost activities managed by the patients themselves, such as support groups. Lymphatic filariasis support groups in Haiti demonstrate the utility of providing patient education in a group setting where members can share their experiences and offer psychosocial support to others coping with similar problems. The Haitian experience highlights the advantages of indigenizing the support group model to enhance participation of affected persons and contribute to the sustainability and autonomy of the groups over time, as well as to community economic development. This experience suggests several features of successful LF support groups: 1) indigenous leadership; 2) flexibility and attentiveness to needs of group members; 3) supportive partners and collaborators; and 4) strategic integration into national LF programs. When adapted to specific cultural and environmental settings, support groups can provide a cost-effective complement to clinical care, particularly for conditions requiring individual self-care at home.
